# Machine learning approach for the prediction of macrosomia

**DOI:** 10.1186/s42492-024-00172-9

**Published:** 2024-08-27

**Authors:** Xiaochen Gu, Ping Huang, Xiaohua Xu, Zhicheng Zheng, Kaiju Luo, Yujie Xu, Yizhen Jia, Yongjin Zhou

**Affiliations:** 1 Eye Hospital, Wenzhou Medical University, Wenzhou, Zheijang 325027 China; 2 School of Biomedical Engineering, Medical School, Shenzhen University, Shenzen, Guangdong 518058 China; 3 Marshall Laboratory of Biomedical Engineering, Shenzen, Guangdong 518058 China; 4 Division of Ultrasound, Department of Medical Imaging, the University of Hong Kong-Shenzhen Hospital, Shenzen, Guangdong 518058 China; 5 Ultrasound Department, the First Affiliated Hospital of Shenzhen University, Second People’s Hospital, Shenzen, Guangdong China 518058; 6 Core Laboratory, the University of Hong Kong-Shenzhen Hospital, Shenzen, Guangdong 518058 China

**Keywords:** Macrosomia, Fetal weight prediction, Machine learning algorithm, Feature selection, Ensemble learning

## Abstract

Fetal macrosomia is associated with maternal and newborn complications due to incorrect fetal weight estimation or inappropriate choice of delivery models. The early screening and evaluation of macrosomia in the third trimester can improve delivery outcomes and reduce complications. However, traditional clinical and ultrasound examinations face difficulties in obtaining accurate fetal measurements during the third trimester of pregnancy. This study aims to develop a comprehensive predictive model for detecting macrosomia using machine learning (ML) algorithms. The accuracy of macrosomia prediction using logistic regression, k-nearest neighbors, support vector machine, random forest (RF), XGBoost, and LightGBM algorithms was explored. Each approach was trained and validated using data from 3244 pregnant women at a hospital in southern China. The information gain method was employed to identify deterministic features associated with the occurrence of macrosomia. The performance of six ML algorithms based on the recall and area under the curve evaluation metrics were compared. To develop an efficient prediction model, two sets of experiments based on ultrasound examination records within 1-7 days and 8-14 days prior to delivery were conducted. The ensemble model, comprising the RF, XGBoost, and LightGBM algorithms, showed encouraging results. For each experimental group, the proposed ensemble model outperformed other ML approaches and the traditional Hadlock formula. The experimental results indicate that, with the most risk-relevant features, the ML algorithms presented in this study can predict macrosomia and assist obstetricians in selecting more appropriate delivery models.

## Introduction

A newborn with a birth weight of 4000 g or more is described by the term macrosomia [1]. Fetal macrosomia can cause multiple maternal and fetal complications. For instance, it can increase the risk of cesarean section for mothers, leading to prolonged labor, labor block, postpartum bleeding, chorioamnionitis, and a higher likelihood of soft birth canal laceration. It also increases the risk of shoulder dystocia, brachial plexus injury, and clavicle fracture in fetuses and newborns. During vaginal delivery, the baseline incidence of shoulder dystocia is 0.2%-3.0%, but when the birth weight reaches 4500 g, the risk of shoulder dystocia dramatically increases to 9%-14% [2]. Given the serious complications involved, timely diagnosis of macrosomia and selection of a more appropriate mode of delivery for pregnant women are clinically significant.

The size and shape of the pelvis are crucial in guiding the choice of delivery model. Owing to factors such as race, nutrition, genetics, and endocrine influences, the morphological structure of the pelvis varies considerably among Chinese and Western women [3]. The pelvises of Western women, such as those from the United States, are often anthropoid. The anteroposterior diameter of an anthropoid pelvis is larger than its lateral diameter, which is a physiological structure that facilitates spontaneous delivery. Approximately half of the pelvises of Chinese women are gynecoid, characterized by a shallow pelvic cavity [4]. If the fetus is overweight or has a large head, the risk of cesarean section significantly increases [5]. Therefore, screening and evaluation of macrosomia in the third trimester are particularly important for Chinese women, as they can improve delivery outcomes and reduce maternal and fetal complications [6, 7]. Among the reports for predicting macrosomia, two broad categories of screening methods are clinical examination and ultrasound assessment [8, 9].

Obstetricians have developed simple formulas to predict fetal weight, which are combined with clinical features, such as maternal abdominal or fundal height [10]. However, factors such as the degree of obesity in pregnant women, abdominal wall thickness, uterine tension, fetal posture, and amniotic fluid depth can lead to significant errors in these formulas when predicting fetal weight, making them insufficiently accurate. Previous studies have indicated that clinical examinations often result in large prediction errors that do not meet clinical requirements [11, 12].

With ongoing advancements in ultrasound equipment and technology, the prediction of fetal weight based on ultrasound measurements of various fetal biological features has become widely utilized. Reports suggest that the ultrasound examination method demonstrates higher accuracy than those of clinical examination methods [13]. Fetal biparietal diameter (BPD), head circumference (HC), abdominal circumference (AC), and femur length (FL) are the most commonly used biological parameters for estimating fetal weight (EFW). Siemer et al. [14] compared the accuracy of 11 widely used EFW formulas and found that the estimation of fetal weight based on Hadlock formulas [15] tends to be more accurate, and multiparameter estimation methods can enhance the precision of EFW. However, these formulas, established by Western scholars, may not account for variations among individuals from different ethnic groups [16]. When applying these methods in China, particularly to large or low-weight fetuses, individual differences among populations can result in significant errors. For instance, the birth weights of only 33%-44% of cases with ultrasound-estimated fetal weights over 4500 g can be accurately predicted [9, 17–21]. Additionally, maternal self-parameters and measurement techniques directly affect the accuracy of EFW. Obtaining precise fetal measurements during the third trimester is often challenging [22], and the absolute error tends to increase with higher estimated fetal weights [19, 21]. Consequently, there is still room for improvement in the current ultrasound examinations used for fetal weight estimation.

Furthermore, machine learning (ML) technologies have previously shown benefits in numerous application domains, including speech recognition, image processing, facial recognition, and automatic diagnosis [23–26]. Additionally, they have been validated for their precision and value in predicting disease outcomes [27]. Consequently, ML techniques have the potential to enhance the efficiency and rationality of decision-making in the prognosis of macrosomia, ultimately aiming to minimize birth defects.

Recently, ML technologies have been recognized and utilized as tools for predicting birth weight. Akhtar et al. [28] conducted a comprehensive study on predicting large for gestational age (LGA) using ML techniques and proposed a support vector machine (SVM) model with a subset of 30 features as the most effective classification model, achieving a precision score of 85% and area under the curve (AUC) of 72%. Their dataset encompassed 220 pilot counties across all 31 provinces of China from 2010 to 2013 [29]. Ye et al. [30] evaluated and compared the accuracies of nonlinear and quadratic mixed-effects models combined with 26 different empirical formulas for EFW. They suggested that ensemble learning could enhance the prediction of LGA. Their datasets were compiled in Norway and Sweden between 1986 and 1988. Lu et al. [31] introduced an ensemble model comprising random forest (RF), XGBoost, and LightGBM algorithms, achieving 64.3% accuracy and 7% mean relative error in predicting fetal weight. Although numerous studies have aimed to predict fetal weight with a certain level of accuracy, there is a paucity of research applying ML techniques with a limited number of features to comprehensively detect macrosomia in pregnant women from southern China.

In this study, a dataset of pregnant women in southern China was established. In addition to the features derived from ultrasound examinations, the clinical characteristics of the pregnant women, including pre-pregnancy body mass index (BMI), gestational weight gain (GWG), fasting blood glucose (FBG), and 2-h postprandial blood glucose (2hPG) were also considered. Subsequently, information gain (IG), a standard univariate filtering method, was used to select the top-ranked features from a pool of 12 [32]. For comparative analysis, six different ML classifiers and the most effective Hadlock formula method [15] were utilized to assess the macrosomia classification performance. However, applying classifiers directly to an imbalanced dataset can significantly affect the experimental outcomes. Therefore, class balancing procedures are essential. An ensemble model was used to refine the results, drawing inspiration from a previous study [31].

## Methods

### Data preprocessing

Data from 3244 pregnant women who delivered between September 2017 and August 2019 at the University of Hong Kong-Shenzhen Hospital were collected and analyzed retrospectively. The dataset was based on electronic health records, which included maternal, fetal, and neonatal clinical features.

Before conducting formal experiments, basic preprocessing steps were implemented. Special cases, such as twins, premature births before 37 weeks, and infants with birth weights below 2500 g, were excluded. Additionally, pregnant women with incomplete records or apparent errors in the clinical data of mothers, fetal parameters, and neonatal outcomes were also excluded. The actual weight data for these fetuses were accurate, with no missing or apparent errors. This study was approved by the Medical Ethics Committee of The University of Hong Kong-Shenzhen Hospital.

To diagnose macrosomia accurately, weighing newborns after birth is essential. In early pregnancy, additional ultrasound examinations do not improve accuracy. A single ultrasound examination during the third trimester is currently the simplest and most effective method for predicting macrosomia [33]. Most birth weight prediction formulas rely primarily on prenatal ultrasound measurements obtained within one week prior to delivery [14, 31, 34]. This study used ultrasound measurements taken within 1-7 days and 8-14 days prior to delivery as input data to establish an efficient prediction model that ensures the accuracy of macrosomia screening before birth. The former aimed to predict macrosomia as comprehensively as possible, while the latter was intended to validate the model.

In this study, we conducted two groups of experiments. The first group utilized six different ML algorithms to classify macrosomia for pregnant women with ultrasound examination records within 1-7 days prior to delivery. Subsequently, the most effective models were combined to form a new model expected to achieve optimal prediction performance. The second experimental setup was designed to validate the model for pregnant women with ultrasound examination records within 8-–14 days before delivery.

In this study, macrosomia refers to infants with a birth weight of 4000 g or more. In the first experiment, 46 infants were classified as macrosomia and 1044 as non-macrosomia, totaling 1090 samples with ultrasound examination records within 1-7 days prior to delivery. The second group comprised 936 samples, including 37 cases of macrosomia and 899 non-macrosomia, with records of ultrasound examinations within 8–14 days prior to delivery. We encoded the actual weight of the newborn and estimated fetal weight using the equation derived by Hadlock et al. [15]. The label was assigned a value of 1 if the actual or estimated weight was 4000 g or more and 0 otherwise.

The dataset included the following 12 features: pre-pregnancy BMI, GWG, in kg, gestational week (GA), gestational diabetes mellitus (GDM), amniotic fluid index (AFI), time interval between the last ultrasound examination and delivery (Interval), FBG, in mmol/L, 2hPG, in mmol/L, fetal AC, in mm, fetal HC, in mm, fetal FL, in mm, and fetal BPD, in mm. Table 1 presents the definitions of each feature.

**Table 1:** Features and their definitions

Feature	Definition
x_2hPG_	2-h postprandial blood glucose (mmol/L)
x_BMI_	Pre-pregnancy body mass index
x_AC_	Fetal abdominal circumference (mm)
x_FBG_	Fasting blood glucose (mmol/L)
x_HC_	Fetal head circumference (mm)
x_FL_	Fetal femur length (mm)
x_GWG_	Gestational weight gain (kg)
x_BPD_	Fetal biparietal diameter (mm)
x_GA_	Gestational week (week)
x_Interval_	Time interval between the last ultrasound examination and delivery (day)
x_GDM_	Gestational diabetes mellitus
x_AFI_	Amniotic fluid index

Feature x_GDM_ has only two values: 0 (non-GDM) and 1 (GDM); Feature x_AFI_ has only three values: -1 (oligohydramnios), 0 (normal amniotic fluid) and 1 (polyhydramnios)

#### Feature standardization

Since different features can have varying units and orders of magnitude, normalizing the data is essential to minimize their impact on the prediction outcomes and ensure that each feature is on a comparable scale. The normalization is shown in Eq. (1):

1 $$\begin{array}{c}y=\frac{x-{x}_{min}}{{x}_{max}-{x}_{min}}\end{array}$$

where $x$ represents the current feature value; ${x}_{min}$ and ${x}_{max}$ represent the minimum and maximum values of the current feature, respectively; and $y$ is the normalized feature value [35]. The data range is [0, 1].

#### Feature selection

Feature selection is a widely utilized technique for identifying features that exhibit a strong correlation with the target class while remaining uncorrelated with other classes. The primary goal of applying feature selection in this study was to develop a classification model that offers enhanced performance and reduced computational overhead. Recently, IG has been employed in various medical domains to screen the top features, yielding positive outcomes [36, 37]. This study adopted IG as a feature selection method to enhance model performance based on these findings.

Generally, this is the difference between the information entropy of the macrosomia dataset *A* with and without feature *t*. There are* L* class labels in dataset *A*, and the information entropy of a class [38] in dataset *A* is denoted by $H\left(A\right)$, which is defined as

2 $$\begin{array}{c}H\left(A\right)=-\sum_{i=1}^nP_{i\;}{\text{log}}_2P_i\end{array}$$

where ${P}_{i}$ is the probability of a labeled class in the macrosomia dataset *A*.

The macrosomia dataset *A* is further divided into *K* groups by feature *t* with *K* different values, namely, ${A}_{k}\left(k=\text{1,2},\dots ,K\right)$. The entropy of each group is calculated as

3 $$\begin{array}{c}H\left(A_k\right)=-\sum_{i=1}^nP_{ki\;}{\text{log}}_2P_{ki}\end{array}$$

where ${P}_{ki}$ defines the probability of a labeled class in subset data ${A}_{k}$ of the basic data *A*. As each group of subset data ${A}_{k}$ contains ${W}_{k}$ samples where ($k=\text{1,2},\dots ,K$), the weight of each group is set to ${W}_{k}$∕$W$. The IG [39] of each feature *t* can be written as

4 $$\begin{array}{c}IG\left(A,t\right)= H\left(A\right)-\sum\limits_{k=1}^{K}\frac{{W}_{k}}{W}H\left({A}_{k}\right)\end{array}$$

Subsequently, the scores generated by IG are sorted in descending order, and the top *i* features are selected as the best variable set for classification.

### ML algorithms

This study aims to address the binary classification problem [40], enabling doctors to detect and diagnose macrosomia as early as possible and provide guidance for delivery methods. ML algorithms offer advantages such as self-training, generalization, self-organization, and learning capabilities. The objective of this study is to develop an effective ML prediction model capable of classifying and predicting macrosomia and non-macrosomia. The performance of the logistic regression (LR), k-nearest neighbors (KNN), SVM, RF, XGBoost, and LightGBM algorithms using the scikit-learn Python toolkit with default parameters were evaluated.

The LR [41] algorithm is a statistical method used for binary classification problems. It estimates the probability of a target variable belonging to a particular class using a logistic function that transforms linear combinations of features into probabilities. The KNN [42] is a simple and effective classification method that assigns a new data point to the class of the majority of its KNN. The SVM classifier [43] is a binary classification method that uses hyperplanes to separate the data points of different classes. It aims to maximize the distance between the hyperplanes and the closest data points of each class, resulting in a robust and accurate classifier. For RF, many regression decision trees are incorporated to improve the accuracy of classification and regression tasks by constructing multiple decision trees and combining their predictive results [44]. It reduces overfitting by randomly sampling data and features and has good generalization ability [45]. XGBoost [46] is an efficient and scalable ML algorithm that uses gradient boosting to build strong predictive models that provide accurate and robust solutions for various classification, regression, and ranking tasks. LightGBM [47] is a gradient-boosting framework that uses efficient parallel training to achieve high performance and low memory consumption. It offers better accuracy and faster training and supports large-scale datasets, making it a versatile tool for ML tasks. LightGBM uses the many-vs-many segmentation method to divide the category features into two subsets to achieve optimal segmentation of the category features. Ensemble methods of creating multiple models in ML are effective prediction methods because they can improve the prediction performance and generalization ability by combining multiple base learners, thereby reducing overfitting and enhancing the classification accuracy [48].

## Performance evaluation indices

The model performance was assessed and compared using two key indicators to determine the optimal prediction model for macrosomia.

The first indicator is Recall, which quantifies the accuracy of correctly identifying true cases of macrosomia. Clinically, a high sensitivity in predicting macrosomia is crucial. The second indicator is the AUC, which provides stable results even with imbalanced datasets. A model with a higher AUC indicates superior performance.

## Results

### Comparison of ML prediction models

As defined above, we executed the experiment using tenfold cross-validation on 1090 cases with ultrasound examination records within 1-7 days prior to delivery and containing 12 features. Table 2 lists the Recall and AUC values of all six ML classifiers and ultrasound estimation based on the Hadlock formula.

**Table 2:** Comparison of Recall and AUC values for six ML methods on 1090 samples with ultrasound examination records 1-7 days before delivery and containing 12 features

Index	Hadlock	LR	SVM	KNN	RF	XGBoost	LightGBM
Recall	0.3650	0.6250	0.8000	0.3350	0.8300	0.8200	0.8050
AUC	0.6787	0.6151	0.7816	0.6034	0.8038	0.8193	0.7940

From the results, we can see that LR, SVM, and KNN performed inefficiently by producing notably low results for Recall and AUC; RF performed best in terms of Recall (0.8300), and XGBoost performed best in terms of AUC (0.8193), whereas LightGBM performed well in both Recall and AUC values. Compared to the ultrasonic estimation, all six ML classifiers performed better. These results demonstrate that ML algorithms may further improve the accuracy of macrosomia screening than that of the Hadlock formula.

### Analysis of feature selection method

**Fig. 1:**
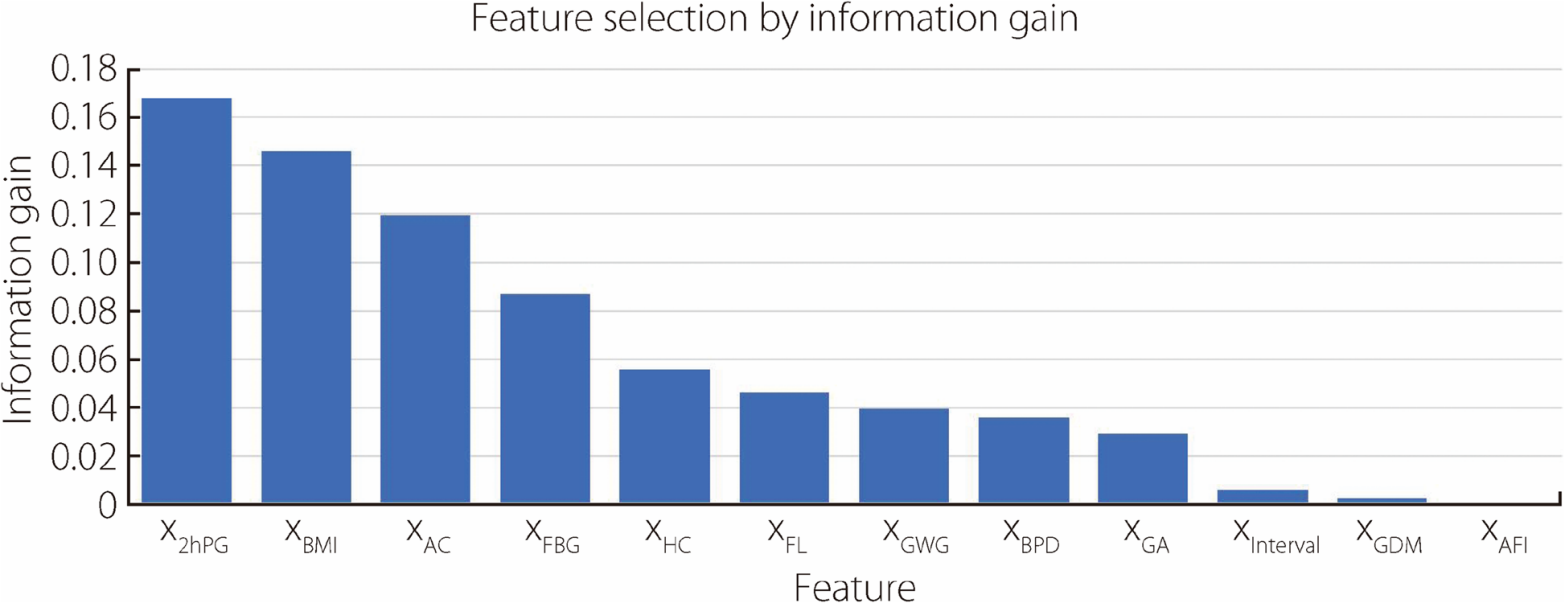
Feature selection using IG

We chose IG as our feature selection method to achieve better performance for each classifier. It ranks the features in descending order based on their high IG entropy. Applying this process, three features with significantly lower IG entropy, namely, x_Interval_, x_GDM_, and x_AFI_, were excluded from the 12 features. The selection results for these features are shown in Fig. 1.

Subsequently, all six ML classifiers were trained using tenfold cross-validation on a dataset that included 1090 cases with nine features for each case. The classification results are listed in Table 3. On comparing the results of Tables 2 and 3, it can be observed that all six ML classifiers demonstrated better prediction performance on the dataset containing nine features than on the dataset containing 12 features. It suggests that the use of the IG method to select features helps improve the prediction performance to some extent.

**Table 3:** Comparison of Recall and AUC values for six ML methods on 1090 samples with ultrasound examination records 1-7 days before delivery and containing 9 features

Index	Hadlock	LR	SVM	KNN	RF	XGBoost	LightGBM
Recall	0.3650	0.7150	0.8250	0.4800	0.8550	0.8600	0.8300
AUC	0.6787	0.7152	0.8278	0.6940	0.8345	0.8375	0.8254

### Analysis of the ensemble model

As previously discussed, we utilized an ensemble model that integrates the top-performing models from Table 3, RF, XGBoost, and LightGBM, to enhance classification performance. This ensemble model was constructed using a voting mechanism.

Subsequently, the three individual models and ensemble model underwent tenfold cross-validation training on a dataset comprising 1090 cases, each featuring nine features, to evaluate their effectiveness in screening for macrosomia. The results of these experiments are presented in Table 4.

**Table 4:** Prediction performance of macrosomia by the ensemble model on 1090 samples with ultrasound examination records 1-7 days before delivery and containing 9 features

Index	Hadlock	RF	XGBoost	LightGBM	Ensemble model
Recall	0.3650	0.8550	0.8600	0.8300	0.8750
AUC	0.6787	0.8345	0.8375	0.8254	0.8460

Table 4 indicates that the ensemble model marginally improved the Recall and AUC values compared to the individual ML algorithms and Hadlock formula. Specifically, Recall showed an improvement of 0.5, while the AUC increased by an estimated 0.17.

### Effectiveness verification of ensemble model

Traditionally, fetal weight estimation has often relied on the Hadlock formula, which is based on multiple ultrasound measurements. However, some fetuses grow rapidly after reaching term, leading to varying ultrasound examination records across different GAs. Consequently, the use of the Hadlock formula may result in significant deviations in the predicted fetal and birth weights owing to the extended time interval between ultrasound examination and delivery [33].

To further validate the ensemble model for predicting fetal weight, it was applied to a dataset comprising ultrasound examination records from 8-14 days before delivery. This dataset includes 936 samples, each featuring nine key features. The classification results are listed in Table 5.

**Table 5:** Prediction performance of macrosomia by the ensemble model on 936 samples with ultrasound examination records 8-14 days before delivery and containing 9 features

Index	Hadlock	RF	XGBoost	LightGBM	Ensemble model
Recall	0.0250	0.7500	0.7333	0.7333	0.7750
AUC	0.5120	0.7482	0.7420	0.7692	0.7585

As shown in Table 5, the ensemble model described in this study demonstrated a notable improvement in Recall and AUC values when compared to the Hadlock formula and the other three ML classifiers. Specifically, Recall and AUC were improved by 0.75 and 0.24, respectively.

## Discussion

There remains scope for advancement in the detection of macrosomia, particularly among pregnant women in southern China. This study focused on pregnant women in southern China who delivered singleton infants at term. We utilized ML algorithms, feeding in maternal and infant features as inputs and EFW labels as outputs. The IG method, grounded in information entropy theory, was employed to evaluate the features that are most predictive of macrosomia. Following feature selection, we conducted two sets of evaluations to assess the prediction performance of macrosomia using ML algorithms. Our approach began with six ML classifiers; the results indicated that RF, XGBoost, and LightGBM performed the best (Table 4). To optimize prediction performance, an ensemble model that integrates the top-performing classifiers (RF, XGBoost, and LightGBM) to predict macrosomia was developed.

### Effective prediction of macrosomia within 1-7 days prior to delivery

The first experimental group, which included ultrasound examination records from days prior to delivery, was used to validate our hypothesis. Table 4 shows that the ensemble model introduced in this study is highly effective for screening macrosomia when the interval between the final ultrasound and delivery is 1-7 days [22]. This ensemble model outperformed the traditional Hadlock formula in terms of predictive accuracy.

### Predicting macrosomia within 8-14 days prior to delivery remains valuable

To verify whether this model was also effective in predicting macrosomia in pregnant women who had ultrasound examination records within 8-–14 days before delivery, we conducted a second set of experiments. As shown in Table 5, the ensemble model remains effective in detecting macrosomia, whereas the Hadlock formula has limitations [16] within this timeframe.

In summary, the ensemble model may be more suitable for screening macrosomia based on ultrasound data obtained 1-2 weeks before delivery. The accurate prediction of macrosomia in the third trimester can encourage pregnant women to be more mindful of their diet and nutritional status, which are crucial for eugenics. Additionally, precise macrosomia assessment enables obstetricians to offer tailored counseling and advice to women at risk of delivering a macrosomic infant, thereby guiding them on appropriate delivery options. Careful consideration of delivery models in clinical practice can reduce the incidence of abnormal deliveries and prevent adverse outcomes for both mothers and infants.

### Ensemble model construction and external validation driven by “1-14 days prior to delivery” data

In clinical practice, a full-term pregnancy is defined as a period of 37 weeks of gestation. Ultrasound examinations are recommended every two weeks during the late stages of pregnancy to ensure the safety of both mother and baby, with particular attention paid to data from the last two weeks before delivery, which is crucial for predicting fetal weight. While previous research has primarily utilized data from 1-7 days before delivery, our inclusion of data from 8-14 days aimed to maximize the capability of the model to detect macrosomia at an earlier stage; accordingly, we categorized the data into two groups: one for the period from 1-7 days before delivery and another for 8-14 days before delivery.

Based on the experimental results, the ensemble model showed significant accuracy in predicting macrosomia. Thus, we applied this model to assess its predictive performance using data from 1-14 days before delivery, with the goal of creating a broadly applicable model for late pregnancy. We aimed to enhance the accuracy of fetal growth assessment and provide robust decision support for clinical practice.

This dataset comprises 2026 cases with ultrasound examination records within 1-14 days prior to delivery, each featuring nine key features. The classification results are listed in Table 6.

**Table 6:** Prediction performance of macrosomia by the ensemble model on 2026 samples with ultrasound examination records 1-14 days before delivery and containing 9 features

Index	Hadlock	RF	XGBoost	LightGBM	Ensemble model
Recall	0.2194	0.7708	0.7944	0.7958	0.8181
Accuracy	0.7640	0.7818	0.8026	0.7793	0.8000
AUC	0.6077	0.7765	0.7987	0.7872	0.8086

Table 6 indicates that the ensemble model described in this study improves the Recall and AUC values to some extent when applied to the dataset with ultrasound examination records within 1-14 days prior to delivery compared to the Hadlock formula and three other ML classifiers. The improvements in Recall and AUC were 0.60 and 0.20, respectively.

**Fig. 2:**
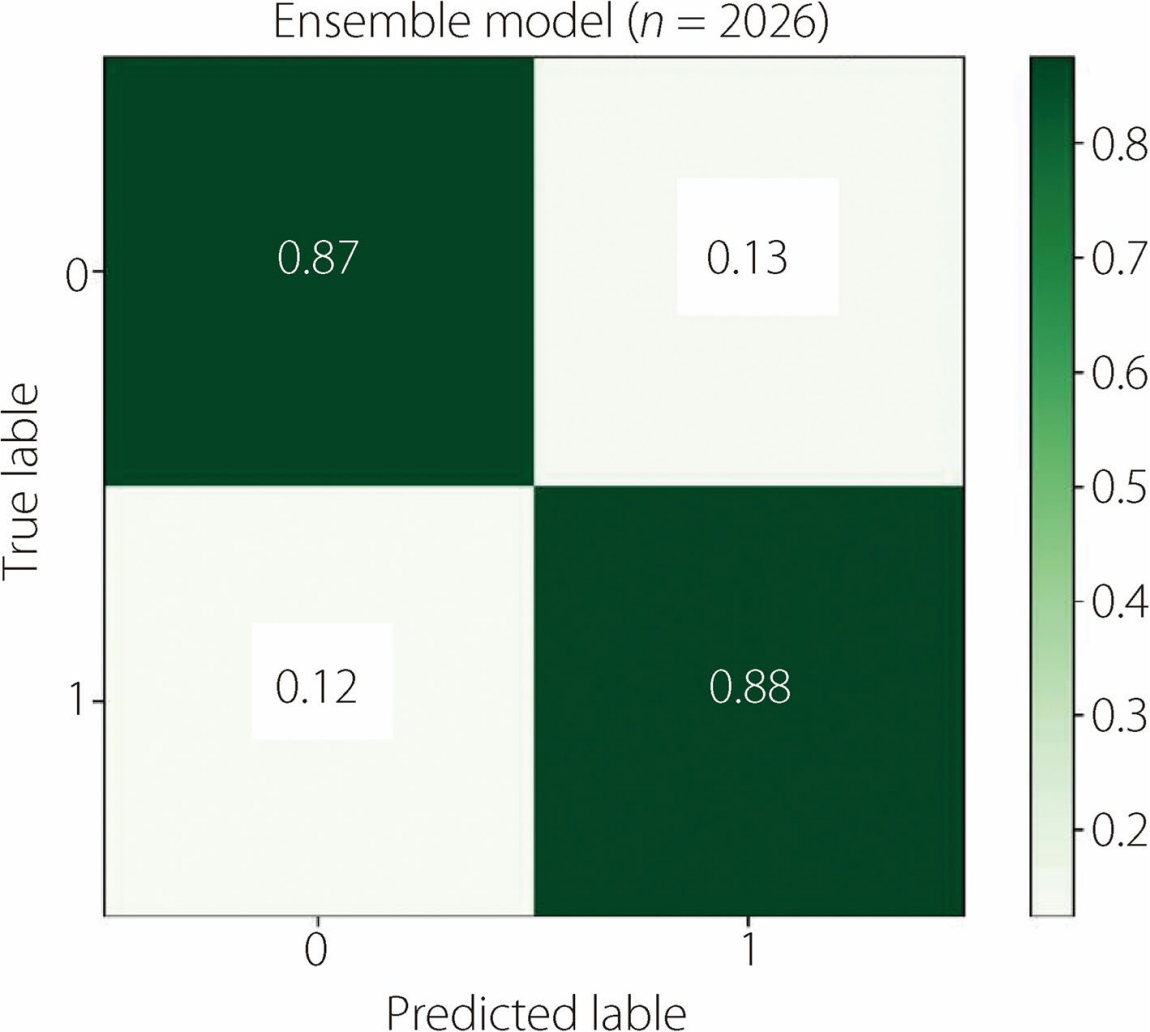
Confusion matrix results

Additionally, to provide a more intuitive illustration of the classification effectiveness of the ensemble model, a confusion matrix was constructed. This matrix features the predicted macrosomia cases along the horizontal axis and actual macrosomia instances along the vertical axis. The displayed confusion matrix has undergone normalization, resulting in the sum of each row (or column) equaling 1, which represents conditional probabilities. The diagonal values within this matrix represent the accurate classification probability for each category, also known as the recall or true positive rate. As shown in Fig. 2, the model exhibits an accuracy rate of 0.88 for predicting macrosomia, which underscores its robust predictive capability.

To comprehensively evaluate the generalization capability of the ensemble model, we additionally collected 44 samples from pregnant women who underwent ultrasound examinations at The Second People’s Hospital of Shenzhen within 1-14 days before delivery, which served as an external validation test set. There were five cases of macrosomia in the sample set. External validation data were reviewed and approved by the Ethics Committee of The Second People’s Hospital of Shenzhen.

The classification results are listed in Table 7. The confusion matrix is shown in Fig. 2. Based on external data validation, it can be seen that the ensemble model has good generalization performance and holds certain clinical significance. The Recall, accuracy, and AUC values were 0.6, 0.77, and 0.75, respectively. The relatively low Recall may be attributed to the small sample size.

**Table 7:** Prediction performance of the ensemble model based on external data

Index	Hadlock	Ensemble model
Recall	0.2000	0.6000
Accuracy	0.5227	0.7727
AUC	0.5350	0.7543

### Predicting low-birth-weight infants is still applicable

It is evident that our model is sensitive to changes in fetal weight; hence, we endeavored to predict another weight extreme: low birth weight (LBW) infants (birth weight under 2500 g [33, 49]). LBW infants exhibit significantly higher morbidity and mortality rates than infants with adequate birth weight [50]. According to Goldenberg and Culhan [50], the mortality rate among LBW infants is 40 times greater than that of normally weighted infants, with an increased likelihood of long-term disabilities. Accurate prenatal fetal weight estimation is crucial for preventing complications related to LBW. Although the Hadlock formula is a widely used clinical tool for fetal weight estimation from ultrasound data, its accuracy is insufficient for LBW infants.

Consequently, an ensemble model combining RF, XGBoost, and LightGBM to predict LBW infant weights, aiming to substantiate the efficacy of the model, was employed.

Initially, cases of twins and macrosomia (birth weight ≥ 4000 g), as well as records with incomplete data or apparent errors, were excluded and then focused on 1128 pregnant women with ultrasound records 1-7 days before delivery, featuring nine key features. Table 8 shows that our ensemble model outperformed the traditional Hadlock formula in predicting LBW infants. Early detection of at-risk pregnancies for LBW infants allows obstetricians to identify fetal growth restrictions and promptly enhance monitoring. This method enables more precise predictions of fetal weight at both extremes, aiding in determining the most suitable delivery method and timing to maximize maternal and fetal safety.

**Table 8:** Prediction performance of LBW infants by the ensemble model on 1128 samples with ultrasound examination records 1-7 days before delivery and containing 9 features

Index	Hadlock	RF	XGBoost	LightGBM	Ensemble model
Recall	0.5417	0.9333	0.9667	0.9333	0.9667
AUC	0.7626	0.9311	0.9260	0.9170	0.9311

### Limitations and future work

Our study has certain limitations that should be considered. Cases with incomplete records or evident errors were excluded from the analysis. This exclusion may have introduced a selection bias because the removed data represented approximately 17% of the entire dataset. In the future, we plan to expand the model by incorporating data from diverse populations to ensure its applicability across various geographical regions, thereby validating the safety and predictability of our prediction model.

## Conclusions

In this study, an ensemble model utilizing data from pregnant women in southern China for the precise screening of macrosomia was introduced. Our findings identified the key determinants of pregnancy that can assist obstetricians in prioritizing and enhancing monitoring. This ensemble model, which integrates RF, XGBoost, and LightGBM, exhibited a high level of reliability in detecting macrosomia in the third trimester. The future application of this model in clinical prenatal care could significantly benefit pre-pregnancy counseling, prenatal evaluation, intrapartum care, postnatal management, and long-term reproductive health.

## Data Availability

Due to the sensitive information contained in our raw data, we cannot provide the raw data.

## Abbreviations

ML: Machine learning
LGA: Large for gestational age
EFW: Estimating fetal weight
2hPG: 2-h postprandial blood glucose
BMI: Body mass index
AC: Abdominal circumference
FBG: Fasting blood glucose
HC: Head circumference
FL: Femur length
GWG: Gestational weight gain
BPD: Biparietal diameter
GA: Gestational week
Interval: Time interval between the last ultrasound examination and delivery
GDM: Gestational diabetes mellitus
AFI: Amniotic fluid index
IG: Information gain
LR: Logistic regression
KNN: K-nearest neighbors
SVM: Support vector machine
RF: Random forest
LBW: Low birth weight
AUC: Area under the curve

## References

[1] Vitner D, Bleicher I, Kadour-Peero E, Lipworth H, Sagi S, Gonen R (2019) Does prenatal identification of fetal macrosomia change management and outcome? Arch Gynecol Obstet 299:635-644. 10.1007/s00404-018-5003-2 30564929 10.1007/s00404-018-5003-2

[2] Gherman RB, Chauhan S, Ouzounian JG, Lerner H, Gonik B, Goodwin TM (2006) Shoulder dystocia: the unpreventable obstetric emergency with empiric management guidelines. Am J Obstet Gynecol 195(3):657-672. 10.1016/j.ajog.2005.09.007 16949396 10.1016/j.ajog.2005.09.007

[3] Abitbol MM (1996) The shapes of the female pelvis. Contributing factors. J Reprod Med 41(4):242-250 8728076

[4] Moloy HC (1960) The use of the roentgen ray in obstetrics. Williams & Wilkins, Baltimore

[5] Caldwell WE, Moloy HC (1933) Anatomical variations in the female pelvis and their effect in labor with a suggested classification. Am J Obstet Gynecol 26(4):479-505. 10.1016/S0002-9378(33)90194-5 10.1016/S0002-9378(33)90194-5

[6] Pressman EK, Bienstock JL, Blakemore KJ, Martin SA, Callan NA (2000) Prediction of birth weight by ultrasound in the third trimester. Obstet Gynecol 95(4):502-506. 10.1097/00006250-200004000-00006 10725480 10.1097/00006250-200004000-00006

[7] Ahmadzia HK, Thomas SM, Dude AM, Grotegut CA, Boyd BK (2014) Prediction of birthweight from third-trimester ultrasound in morbidly obese women. Am J Obstet Gynecol 211(4):431.e1-431.e7. 10.1016/j.ajog.2014.06.041 10.1016/j.ajog.2014.06.041 24954654

[8] Chauhan SP, Cowan BD, Magann EF, Bradford TH, Roberts WE, Morrison JC (1995) Intrapartum detection of a MAEROSOMIE fetus: clinial versus 8 sonographic models. Aust N Z J Obstet Gynaecol 35(3):266-270. 10.1111/j.1479-828X.1995.tb01978.x 8546640 10.1111/j.1479-828X.1995.tb01978.x

[9] Chauhan SP, Hendrix NW, Magann EF, Morrison JC, Kenney SP, Devoe LD (1998) Limitations of clinical and sonographic estimates of birth weight: experience with 1034 parturients. Obstet Gynecol 91(1):72-77. 10.1016/S0029-7844(97)00590-5 9464724 10.1016/S0029-7844(97)00590-5

[10] Kayem G, Grangé G, Bréart G, Goffinet F (2009) Comparison of fundal height measurement and sonographically measured fetal abdominal circumference in the prediction of high and low birth weight at term. Ultrasound Obstet Gynecol 34(5):566-571. 10.1002/uog.6378 19582801 10.1002/uog.6378

[11] Anderson NG, Jolley IJ, Wells JE (2007) Sonographic estimation of fetal weight: comparison of bias, precision and consistency using 12 different formulae. Ultrasound Obstet Gynecol 30(2):173-179. 10.1002/uog.4037 17557378 10.1002/uog.4037

[12] Scioscia M, Scioscia F, Scioscia G, Bettocchi S (2015) Statistical limits in sonographic estimation of birth weight. Arch Gynecol Obstet 291(1):59-66. 10.1007/s00404-014-3384-4 25069646 10.1007/s00404-014-3384-4

[13] Lanowski JS, Lanowski G, Schippert C, Drinkut K, Hillemanns P, Staboulidou I (2017) Ultrasound versus clinical examination to estimate fetal weight at term. Geburtshilfe Frauenheilkd 77(3):276-283. 10.1055/s-0043-102406 28392581 10.1055/s-0043-102406 PMC5383430

[14] Siemer J, Egger N, Hart N, Meurer B, Müller A, Dathe O et al (2008) Fetal weight estimation by ultrasound: comparison of 11 different formulae and examiners with differing skill levels. Ultraschall Med 29(2):159-164. 10.1055/s-2007-963165 17602369 10.1055/s-2007-963165

[15] Hadlock FP, Harrist RB, Carpenter RJ, Deter RL, Park SK (1984) Sonographic estimation of fetal weight. The value of femur length in addition to head and abdomen measurements. Radiology 150(2):535–540. 10.1148/radiology.150.2.6691115 10.1148/radiology.150.2.6691115 6691115

[16] Bowers K, Laughon SK, Kiely M, Brite J, Chen Z, Zhang C (2013) Gestational diabetes, pre-pregnancy obesity and pregnancy weight gain in relation to excess fetal growth: variations by race/ethnicity. Diabetologia 56(6):1263-1271. 10.1007/s00125-013-2881-5 23571827 10.1007/s00125-013-2881-5 PMC10440833

[17] Malin GL, Bugg GJ, Takwoingi Y, Thornton JG, Jones NW (2016) Antenatal magnetic resonance imaging versus ultrasound for predicting neonatal macrosomia: a systematic review and meta-analysis. BJOG 123(1):77-88. 10.1111/1471-0528.13517 26224221 10.1111/1471-0528.13517

[18] Scioscia M, Vimercati A, Ceci O, Vicino M, Selvaggi LE (2008) Estimation of birth weight by two-dimensional ultrasonography: a critical appraisal of its accuracy. Obstet Gynecol 111(1):57-65. 10.1097/01.AOG.0000296656.81143.e6 18165393 10.1097/01.AOG.0000296656.81143.e6

[19] Zafman KB, Bergh E, Fox NS (2020) Accuracy of sonographic estimated fetal weight in suspected macrosomia: the likelihood of overestimating and underestimating the true birthweight. J Matern Fetal Neonatal Med 33(6):967-972. 10.1080/14767058.2018.1511697 30099910 10.1080/14767058.2018.1511697

[20] Sandmire HF (1993) Whither ultrasonic prediction of fetal macrosomia? Obstet Gynecol 82(5):860-862 8414339

[21] Aviram A, Yogev Y, Ashwal E, Hiersch L, Danon D, Hadar E et al (2017) Different formulas, different thresholds and different performance-the prediction of macrosomia by ultrasound. J Perinatol 37(12):1285-1291. 10.1038/jp.2017.134 28906497 10.1038/jp.2017.134

[22] Milner J, Arezina J (2018) The accuracy of ultrasound estimation of fetal weight in comparison to birth weight: A systematic review. Ultrasound 26(1):32-41. 10.1177/1742271X17732807 29456580 10.1177/1742271X17732807 PMC5810856

[23] Fröhlich H, Claes K, De Wolf C, Van Damme X, Michel A (2017) A machine learning approach to automated gait analysis for the Noldus Catwalk System. IEEE Trans Biomed Eng 65(5):1133-1139. 10.1109/TBME.2017.2701204 28858780 10.1109/TBME.2017.2701204

[24] Ding CX, Tao DC (2018) Trunk-branch ensemble convolutional neural networks for video-based face recognition. IEEE Trans Pattern Anal Mach Intellig 40(4):1002-1014. 10.1109/TPAMI.2017.2700390 10.1109/TPAMI.2017.2700390 28475048

[25] Wong KKL, Wang LS, Wang DF (2017) Recent developments in machine learning for medical imaging applications. Comput Med Imaging Graph 57:1-3. 10.1016/j.compmedimag.2017.04.001 28456276 10.1016/j.compmedimag.2017.04.001

[26] Zhang Q, Hansen JHL (2018) Language/dialect recognition based on unsupervised deep learning. IEEE/ACM Trans Audio Speech Language Process 26(5):873-882. 10.1109/TASLP.2018.2797420 10.1109/TASLP.2018.2797420

[27] Naimi AI, Platt RW, Larkin JC (2018) Machine learning for fetal growth prediction. Epidemiology 29(2):290-298. 10.1097/EDE.0000000000000788 29199998 10.1097/EDE.0000000000000788 PMC5792310

[28] Akhtar F, Li JQ, Azeem M, Chen S, Pan H, Wang Q et al (2019) Effective large for gestational age prediction using machine learning techniques with monitoring biochemical indicators. J Supercomput 76(8):6219-6237. 10.1007/s11227-018-02738-w 10.1007/s11227-018-02738-w

[29] Zhang SK, Wang QM, Shen HP (2015) Design implementation and significance of Chinese free pre-pregnancy eugenics checks project. Nat Med J China 95(3):162-165

[30] Ye SY, Zhang H, Shi FY, Guo J, Wang SZ, Zhang B (2020) Ensemble learning to improve the prediction of fetal macrosomia and large-for-gestational age. JCM 9(2):380. 10.3390/jcm9020380 32023935 10.3390/jcm9020380 PMC7074295

[31] Lu Y, Fu XH, Chen FX, Wong KKL (2020) Prediction of fetal weight at varying gestational age in the absence of ultrasound examination using ensemble learning. Artif Intellig Med 102:101748. 10.1016/j.artmed.2019.101748 10.1016/j.artmed.2019.101748 31980089

[32] Li JQ, Liu L, Sun JC, Mo HW, Yang JJ, Chen S et al (2020) Comparison of different machine learning approaches to predict small for gestational age infants. IEEE Trans Big Data 6(2):334-346. 10.1109/TBDATA.2016.2620981 10.1109/TBDATA.2016.2620981

[33] Zhang J, Kim S, Grewal J, Albert PS (2012) Predicting large fetuses at birth: do multiple ultrasound examinations and longitudinal statistical modelling improve prediction? Paediatr Perinatal Epidemiol 26(3):199-207. 10.1111/j.1365-3016.2012.01261.x 10.1111/j.1365-3016.2012.01261.x PMC3324111 22471679

[34] Peregrine E, O’Brien P, Jauniaux E (2007) Clinical and ultrasound estimation of birth weight prior to induction of labor at term. Ultrasound Obstet Gynecol 29(3):304–309. 10.1002/uog.3949 17290365 10.1002/uog.3949

[35] Patro SGK, Sahu KK (2015) Normalization: a preprocessing stage. Int Adv Res J Sci Eng Technol 2(3):20–22. 10.17148/IARJSET.2015.2305

[36] Kullback S, Leibler RA (1951) On information and sufficiency. Ann Math Stat 22(1):79-86. 10.1214/aoms/1177729694 10.1214/aoms/1177729694

[37] Raju R (2012) Relative importance of fine needle aspiration features for breast cancer diagnosis: a study using information gain evaluation and machine learning. J Am Soc Cytopathol 1(Suppl 1):S11. 10.1016/j.jasc.2012.08.017 10.1016/j.jasc.2012.08.017

[38] Shannon CE. A mathematical theory of communication. ACM SIGMOBILE Mobile Comput Commun Rev 5(1):3–55. 10.1145/584091.584093

[39] Azhagusundari B, Thanamani AS (2013) Feature selection based on information gain. Int J Innovat Technol Explor Eng 2(2):18-21

[40] Khashei M, Eftekhari S, Parvizian J (2012) Diagnosing diabetes type II using a soft intelligent binary classification model. Rev Bioinformat Biomet 1(1):9-23

[41] Freedman DA (2009) Statistical models: theory and practice. Cambridge University Press, Cambridge. 10.1017/CBO9780511815867 10.1017/CBO9780511815867

[42] Guo GD, Wang H, Bell D, Bi YX, Greer K (2003) KNN model-based approach in classification. In: Meersman R, Tari Z, Schmidt D C (eds) On the move to meaningful internet systems 2003: CoopIS, DOA, and ODBASE. OTM confederated international conferences CoopIS, DOA, and ODBASE 2003 Catania, Sicily, Italy, November 2003. Lecture notes in computer science, vol 2888. Springer, Sicily, pp 986–996. 10.1007/978-3-540-39964-3_62

[43] Hearst MA, Dumais ST, Osuna E, Platt J, Scholkopf B (1998) Support vector machines. IEEE Intell Syst Their Appl 13(4):18-28. 10.1109/5254.708428 10.1109/5254.708428

[44] Berk RA (2020) Statistical learning from a regression perspective. Springer, Cham. 10.1007/978-3-030-40189-4 10.1007/978-3-030-40189-4

[45] Breiman L (2001) Random forests. Machine Learning 45(1):5-32. 10.1023/A:1010933404324 10.1023/A:1010933404324

[46] Chen TQ, Guestrin C (2016) XGBoost: A scalable tree boosting system. In: Proceedings of the 22nd ACM SIGKDD international conference on knowledge discovery and data mining, ACM, San Francisco, 13 August 2016. 10.1145/2939672.2939785

[47] Ke GL, Meng Q, Finley T, Wang TF, Chen W, Ma W et al (2017) LightGBM: A highly efficient gradient boosting decision tree. In: Proceedings of the 31st international conference on neural information processing systems, Curran Associates Inc., Long Beach, 4 December 2017

[48] Dietterich TG (2000) An experimental comparison of three methods for constructing ensembles of decision trees: bagging, boosting, and randomization. Machine Learning 40(2):139-157. 10.1023/A:1007607513941 10.1023/A:1007607513941

[49] Das USG, Sysyn GD (2004) Abnormal fetal growth: intrauterine growth retardation, small for gestational age, large for gestational age. Pediatr Clin North Am 51(3):639-654. 10.1016/j.pcl.2004.01.004 15157589 10.1016/j.pcl.2004.01.004

[50] Goldenberg RL, Culhane JF (2007) Low birth weight in the United States. Am J Clin Nutr 85(2):584S-590S. 10.1093/ajcn/85.2.584S 17284760 10.1093/ajcn/85.2.584S

